# Relative Importance and Additive Effects of Maternal and Infant Risk Factors on Childhood Asthma

**DOI:** 10.1371/journal.pone.0151705

**Published:** 2016-03-22

**Authors:** Pingsheng Wu, Amy S. Feldman, Christian Rosas-Salazar, Kristina James, Gabriel Escobar, Tebeb Gebretsadik, Sherian Xu Li, Kecia N. Carroll, Eileen Walsh, Edward Mitchel, Suman Das, Rajesh Kumar, Chang Yu, William D. Dupont, Tina V. Hartert

**Affiliations:** 1 Division of Allergy, Pulmonary, and Critical Care Medicine, Department of Medicine, and Center for Asthma and Environmental Sciences Research, Vanderbilt University School of Medicine, Nashville, Tennessee, United States of America; 2 Department of Biostatistics, Vanderbilt University School of Medicine, Nashville, Tennessee, United States of America; 3 Department of Pediatrics, Vanderbilt University School of Medicine, Nashville, Tennessee, United States of America; 4 Peninsula Allergy & Asthma Center, Soldotna, Alaska, United States of America; 5 Kaiser Permanente Medical Care Program, Oakland, California, United States of America; 6 Kaiser Permanente Northern California, Perinatal Research Unit, Division of Research, Oakland, California, United States of America; 7 Department of Health Policy, Vanderbilt University School of Medicine, Nashville, Tennessee, United States of America; 8 J. Craig Venter Institute, Rockville, Maryland, United States of America; 9 The Ann and Robert H. Lurie Children’s Hospital of Chicago and Northwestern University, Chicago, Illinois, United States of America; National Institute of Health, ITALY

## Abstract

**Background:**

Environmental exposures that occur *in utero* and during early life may contribute to the development of childhood asthma through alteration of the human microbiome. The objectives of this study were to estimate the cumulative effect and relative importance of environmental exposures on the risk of childhood asthma.

**Methods:**

We conducted a population-based birth cohort study of mother-child dyads who were born between 1995 and 2003 and were continuously enrolled in the PRIMA (***P****revention of* ***R****SV*: ***I****mpact on* ***M****orbidity and* ***A****sthma)* cohort. The individual and cumulative impact of maternal urinary tract infections (UTI) during pregnancy, maternal colonization with group B streptococcus (GBS), mode of delivery, infant antibiotic use, and older siblings at home, on the risk of childhood asthma were estimated using logistic regression. Dose-response effect on childhood asthma risk was assessed for continuous risk factors: number of maternal UTIs during pregnancy, courses of infant antibiotics, and number of older siblings at home. We further assessed and compared the relative importance of these exposures on the asthma risk. In a subgroup of children for whom maternal antibiotic use during pregnancy information was available, the effect of maternal antibiotic use on the risk of childhood asthma was estimated.

**Results:**

Among 136,098 singleton birth infants, 13.29% developed asthma. In both univariate and adjusted analyses, maternal UTI during pregnancy (odds ratio [OR] 1.2, 95% confidence interval [CI] 1.18, 1.25; adjusted OR [AOR] 1.04, 95%CI 1.02, 1.07 for every additional UTI) and infant antibiotic use (OR 1.21, 95%CI 1.20, 1.22; AOR 1.16, 95%CI 1.15, 1.17 for every additional course) were associated with an increased risk of childhood asthma, while having older siblings at home (OR 0.92, 95%CI 0.91, 0.93; AOR 0.85, 95%CI 0.84, 0.87 for each additional sibling) was associated with a decreased risk of childhood asthma, in a dose-dependent manner. Compared with vaginal delivery, C-section delivery increased odds of childhood asthma by 34% (OR 1.34, 95%CI 1.29, 1.39) in the univariate analysis and 11% after adjusting for other environmental exposures and covariates (AOR 1.11, 95%CI 1.06, 1.15). Maternal GBS was associated with a significant increased risk of childhood asthma in the univariate analysis (OR 1.27, 95%CI 1.19, 1.35), but not in the adjusted analysis (AOR 1.03, 95%CI 0.96, 1.10). In the subgroup analysis of children whose maternal antibiotic use information was available, maternal antibiotic use was associated with an increased risk of childhood asthma in a similar dose-dependent manner in the univariate and adjusted analyses (OR 1.13, 95%CI 1.12, 1.15; AOR 1.06, 95%CI 1.05, 1.08 for every additional course). Compared with infants with the lowest number of exposures (no UTI during pregnancy, vaginal delivery, at least five older siblings at home, no antibiotics during infancy), infants with the highest number of exposures (at least three UTIs during pregnancy, C-section delivery, no older siblings, eight or more courses of antibiotics during infancy) had a 7.77 fold increased odds of developing asthma (AOR: 7.77, 95%CI: 6.25, 9.65). Lastly, infant antibiotic use had the greatest impact on asthma risk compared with maternal UTI during pregnancy, mode of delivery and having older siblings at home.

**Conclusion:**

Early-life exposures, maternal UTI during pregnancy (maternal antibiotic use), mode of delivery, infant antibiotic use, and having older siblings at home, are associated with an increased risk of childhood asthma in a cumulative manner, and for those continuous variables, a dose-dependent relationship. Compared with *in utero* exposures, exposures occurring during infancy have a greater impact on the risk of developing childhood asthma.

## Introduction

Asthma is the most common serious chronic disease of childhood [1]. The worldwide prevalence of asthma has increased during the past 30 years [2]. Increasing evidence suggests that the human microbiome plays an important role in the development of asthma [3].

The human microbiome is the collective genomes and gene products of microbes living in and on the human body [4,5]. It plays an important role in the development of protective immune responses [6]. The alteration of the microbiome, particularly during the prenatal, perinatal, and infant period, may lead to the development of asthma and atopic diseases [7–10]. Numerous studies have shown that exposures which modify the early-life microbiome (such as prenatal and postnatal antibiotic use [11–15], mode of delivery [16–22], early diet [23–34], and number of older siblings [35,36]) are associated with an increased risk of childhood asthma. However, the relative, dose-dependent and cumulative impact of these early-life exposures on the development of asthma is not known. Neither do we know which early-life exposures have the most influence on asthma inception, nor whether prenatal and postnatal exposures contribute equally. The objective of this study was to determine the relative impact of and cumulative effect of *in utero*, perinatal, and postnatal exposures that could be measured during pregnancy and infancy on the risk of developing early childhood asthma: maternal antibiotic use/urinary tract infection (UTI), mode of delivery, infant antibiotic use, and having older siblings. We also assessed the dose-dependent relationship of maternal antibiotic use/UTI, infant antibiotic use, and number of older siblings on the risk of developing early childhood asthma.

## Materials and Methods

### Study population

The study population is part of an existing large population-based birth cohort study, PRIMA (***P****revention of* ***R****SV*: ***I****mpact on* ***M****orbidity and* ***A****sthma)* [37]. We included singleton birth mother-child dyads born between 1995–2003, birth weight of 500–6999 grams, gestational age between 24 0/7 and 45 5/7 weeks, and with continuous enrollment and birth in one of six Kaiser Permanente Northern California (KPNC) hospitals or enrollment and birth in the Tennessee Medicaid program (TennCare). We required continuous enrollment for both mothers and children. For mothers, continuous enrollment was defined as 60 or fewer days of disenrollment in either program during the period from 180 days before last menstrual period to date of delivery. For children, we defined continuous enrollment as 90 or fewer days of disenrollment in either program during the period from birth to 365 days of age and age 4.5 to 6 years. KPNC and TennCare provide health insurance for approximately 30% of all the deliveries and births each year among the insured population of Northern California and 50% in Tennessee, respectively. The rationale and detailed methods for the PRIMA cohort have been reported previously [37]. The study protocol was approved by the KPNC Institutional Board for the Protection of Human Subjects, the State of California Committee for the Protection of Human Subjects, and the Vanderbilt University Institutional Review Board.

### Event ascertainment

Events or exposures during pregnancy, at delivery, and during infancy, included number of maternal urinary tract infections (UTI) during pregnancy, maternal colonization with group B streptococcus (GBS), mode of delivery, antibiotic use during infancy, and number of older siblings at home. Maternal UTI during pregnancy and maternal GBS captured using ICD-9-CM codes served as our indicator of maternal infection and antibiotic use during pregnancy. Mode of delivery (vaginal, assisted, and C-section delivery) and number of older siblings were determined from the linked birth certificates. Infant antibiotic use during the first 12 months of life was captured from medical claims prescription fill data of TennCare or electronic medical record data of KPNC. Lastly, we also directly captured maternal antibiotic use during pregnancy in subjects enrolled in TennCare.

### Childhood asthma ascertainment

Childhood asthma was ascertained between age 4.5 to 6 years using healthcare encounters and pharmacy claims data. Children with an ICD-9-CM diagnosis of 493 in any diagnostic field for inpatient, other hospital care, emergency department visit, and/or outpatient physician visit claims (two outpatient visits separated by at least 30 days were required) were considered to have asthma. In addition, children with two prescriptions for any short-acting beta-agonist, two prescriptions for montelukast in a 365 day period prior to Food and Drug Administration approval of montelukast for allergic rhinitis, or a single prescription of any other asthma-specific medication were considered as having asthma. Our algorithm for asthma ascertainment has been previously validated and is similar to that used by other US Medicaid systems [38,39]. We identified asthma at age 4.5 years to 6 years to exclude the transient wheezing phenotype. Furthermore, we allowed 18 months ascertainment period to capture mild asthmatics [38].

### Covariates

Maternal smoking during pregnancy, maternal asthma status, maternal age at delivery, maternal education level, gestational age at delivery, infant’s birth hospitalization length of stay, birth weight, infant’s race, gender, having chronic lung disease, having congenital heart disease, type of most severe bronchiolitis healthcare encounters experienced during infancy (hospitalization, emergence department visit, outpatient visit, and no visit), and birth year were determined from medical records or linked birth certificates. We determined maternal asthma status using a similar algorithm defined for childhood asthma (see above). All covariates, including study site (KPNC or TennCare) were chosen *a priori* based on clinical relevance.

### Statistical analysis

We expressed descriptive statistics as frequencies and proportions for categorical variables, and as means and standard deviations (SD) or medians and interquartile ranges (IQR) for continuous variables, depending on the distribution of the variables.

To assess the relationship of early life exposures with the risk of childhood asthma, we performed univariate and multivariable logistic regression models. The main exposures of interest included pregnancy exposure (number of maternal UTI during pregnancy: 0 1, 2, and 3+), delivery exposures (GBS: yes/no; and mode of delivery: vaginal, assisted, and C-section delivery), infant exposures (antibiotic use: 0, 1, 2, 3, 4, 5, 6, 7, and 8+; and number of older siblings: 0, 1, 2, 3, 4, and 5+). Covariates listed above were adjusted in the multivariable regression models. Maternal UTI during pregnancy, infant antibiotic use, and number of older siblings were treated as categorical variables to estimate the specific effect at each dose level, as well as continuous variables to estimate the dose response effect. Relative contribution and the importance of each exposure on the asthma risk were assessed by the proportion of asthma outcome variability explained by each individual exposure on the Chi-square scale [40].

In children whose mothers were continuously enrolled in TennCare where specific maternal antibiotic prescription dispensing information was available, we directly estimated the effect of maternal antibiotic use during pregnancy on the risk of childhood asthma.

As prematurity and low birth weight are often associated with use of systemic antibiotics in practice. We conducted a subset analysis of term (≥37 weeks), non-low birth weight (≥2500 grams), and healthy (no chronic lung disease and no congenital heart disease) infants to evaluate the association between exposures of interest and childhood asthma.

Lastly, instead of using maternal UTI during pregnancy as markers of maternal infection and antibiotic use, we captured any type of infection and conducted a sensitivity analysis to determine if we could distinguish the effect of maternal infection during pregnancy versus maternal antibiotics on the risk of childhood asthma. Similar analyses using infant infection instead of infant antibiotic use were conducted. All above analyses were repeated separately for each study site.

All analyses were performed using R-software version 3.1.2 (www.r-project.org) [41] package rms [40] and data management using SAS version 9.4 (SAS Institutes; Cary, NC). We used a two-sided 5% significance level for all statistical analyses.

## Results

There were 136,098 singleton birth mother-child dyads (33,142 from KPNC and 102,956 from TennCare) in this study. There were 18,081 (13.29%) infants (3,075 [9.28%] from KPNC and 15,006 [14.58%] from TennCare) who developed asthma at age 6 years. The average mothers’ age at delivery was 25 (SD: 6.34) years. The average gestational age was 39 (SD: 2.37) weeks and the average birth weight was 3,221 (SD: 603) grams. Compared with children who enrolled in KPNC, children enrolled in TennCare were more likely to be born prematurely, be low birth weight, have at least one infant bronchiolitis hospitalization, have a younger mother, have a mother who smoked during pregnancy, and have a less educated mother (p<0.001 for all comparisons) (Table 1).

**Table 1:** Maternal and infant characteristics by type of healthcare coverage among children enrolled in PRIMA cohort, 1995–2009 (n = 136,098).

Characteristics	KPNC 33,142 (24.35%)	TennCare 102,956 (75.65%)
Maternal age at delivery (years) (n = 135,938), median (IQR^)*	31.00 (27.00, 35.00)	22.00 (19.00, 26.00)
Maternal smoking during pregnancy (n = 135,866), n (%)*	1,324 (3.99)	29,124 (28.29)
Maternal education (years) (n = 134,973), n (%)*		
<12	2,610 (7.88)	48,229 (46.84)
12	7,663 (23.12)	44,269 (43.00)
>12	21,972 (66.30)	10,230 (9.94)
Maternal asthma, n (%)	1,518 (4.58)	4,628 (4.50)
Gestational age (weeks), median (IQR)*	39.00 (38.00, 40.00)	39.00 (38.00, 40.00)
Infant birth weight (grams), median (IQR)*	3445.00 (3105.00, 3788.00)	3200.50 (2835.00, 3515.00)
Infant male gender (n = 136,091), n (%)	16,992 (51.27)	52,772 (51.26)
Infant race (n = 136,047), n (%)*		
White	14586 (44.01)	50,244 (48.80)
Black	2,025 (6.11)	46,734 (45.40)
Hispanic	6,280 (18.95)	1,041 (1.01)
Asian	7,810 (23.57)	600 (0.58)
Other	2,390 (7.21)	4,334 (4.21)
One or more infant bronchiolitis hospitalization(s), n (%)*	4245 (12.81)	22815 (22.16)

^IQR: interquartile range

*p <0.001

There were 18,834 (13.84%) infants whose mothers had at least one UTI during pregnancy; 8,016 (6%) infants whose mothers had GBS at delivery, and 28,945 (21%) born via C-section. Thirty three percent (44,721) of infants had no older siblings at home, and 73% (99,054) of infants had at least one course of antibiotics during the first year of life (Table 2).

**Table 2:** *In utero* and early life exposures by type of healthcare coverage among children enrolled in the PRIMA cohort, 1995–2009 (n = 136,098).

Characteristics	KPNC n = 33,142 (24.35%)	TennCare n = 102,956 (75.65%)
***In utero* and delivery exposures**		
Maternal UTI during pregnancy, n (%)		
0	31,984 (96.51)	85,280 (82.83)
1	1,034 (3.12)	12,947 (12.58)
2	104 (0.31)	3,151 (3.06)
≥3	20 (0.06)	1,578 (1.53)
Maternal GBS, n (%)		
No	31,934 (96.36)	96,148 (93.39)
Yes	1,208 (3.64)	6,808 (6.61)
Mode of delivery (n = 136,060), n (%)		
Vaginal	24,147 (72.86)	73,644 (71.53)
Assisted	2,159 (6.51)	7,165 (6.96)
C-section	6,836 (20.63)	22,109 (21.47)
**Infant exposures**		
Infant antibiotics use, n (%)		
0	16,328 (49.27)	20,716 (20.12)
1	8,774 (26.47)	21,640 (21.02)
2	4,392 (13.25)	19,285 (18.73)
3	2,148 (6.48)	15,010 (14.58)
4	920 (2.78)	10,650 (10.34)
5	382 (1.15)	7,104 (6.90)
6	122 0.37)	4,251 (4.13)
7	44 (0.13)	2,300 (2.23)
≥8	32 (0.10)	2,000 (1.94)
Number of older siblings (n = 136,011), n (%)		
0	13,539 (40.85)	31,182 (30.29)
1	12,300 (37.11)	35,596 (34.57)
2	5,028 (15.17)	20,413 (19.83)
3	1,571 (4.74)	8,947 (8.69)
4	426 (1.29)	3,638 (3.53)
≥5	273 (0.82)	3,098 (3.01)

### *In utero* and delivery exposures

**Fig 1:**
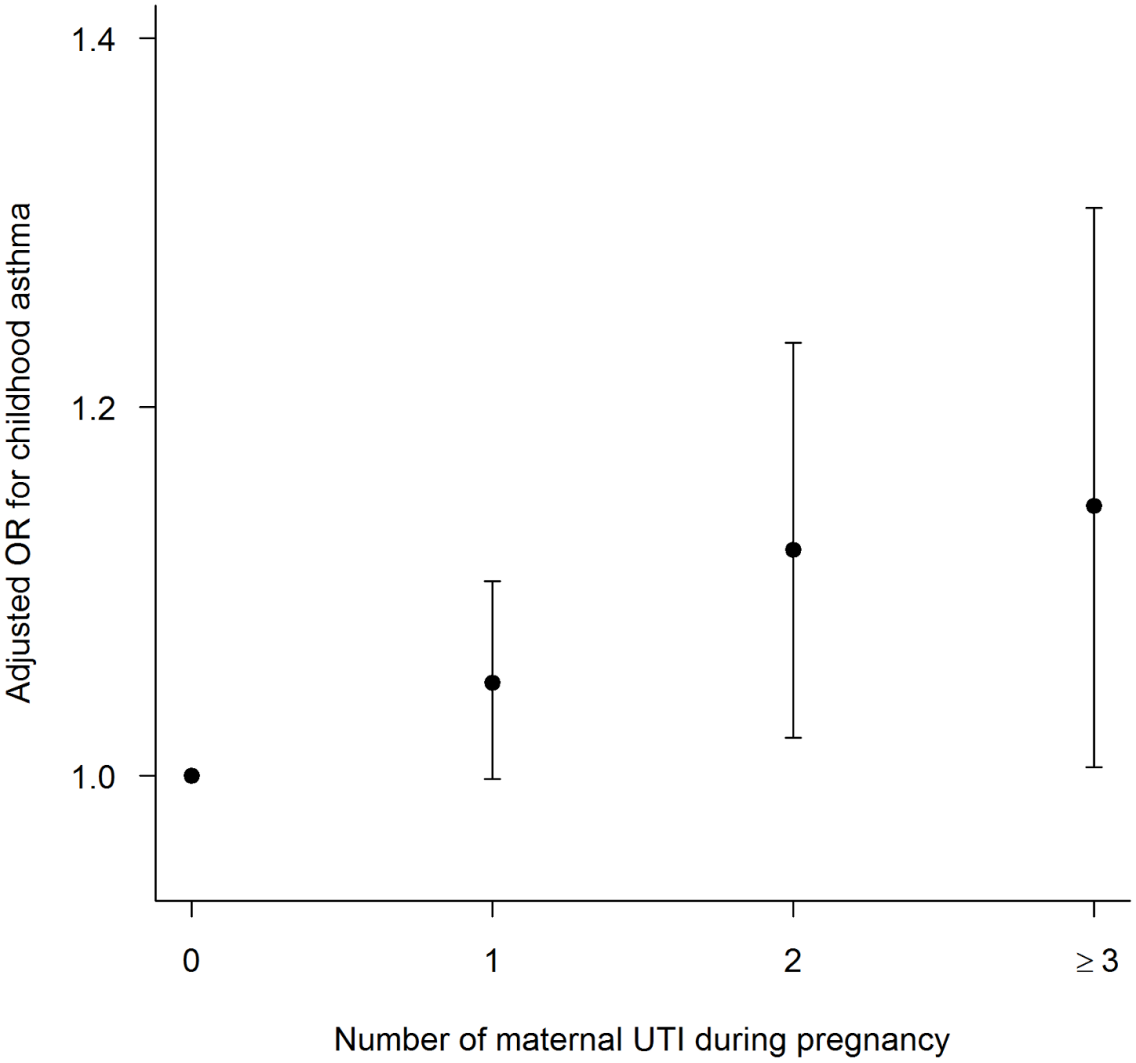
Adjusted odds ratio (AOR) for childhood asthma in relation to the number of maternal UTIs during pregnancy. Infants of mothers who did not have a UTI during pregnancy served as the reference group.

Maternal UTI during pregnancy significantly increased the risk of childhood asthma in both unadjusted and adjusted analyses. Compared with children whose mothers did not have a UTI during pregnancy, having at least one UTI during pregnancy increased the odds of having childhood asthma by 34% (Odds ratio [OR] 1.34, 95% confidence interval [CI] 1.28, 1.40). There was a significant dose-dependent relationship between increasing number of maternal UTIs and the risk of asthma development. For every additional prenatal UTI women experienced, the odds of their children developing asthma increased by 20% (OR 1.20, 95%CI 1.18, 1.25). The significant dose-dependent association between maternal UTI during pregnancy and the risk of childhood asthma persisted after adjusting for maternal GBS, mode of delivery, infant antibiotic use, number of older siblings at home, and other confounding covariates (Adjusted OR [AOR] 1.04, 95%CI 1.02, 1.07) (Fig 1). In the analysis with any type of maternal infection during pregnancy included, maternal infection increased the odds of childhood asthma in a similar dose response manner (one episode of infection: AOR 1.06, 95%CI 1.04, 1.08) (S1 Fig).

GBS colonization at delivery, the least common of all exposures, increased the odds of childhood asthma by 27% (OR 1.27, 95%CI 1.19, 1.35) in the unadjusted analysis. However, the association was not significant after adjusting for other exposures and confounding covariates (AOR 1.03, 95%CI 0.96, 1.10). C-section was associated with an increased risk of childhood asthma in both univariate and multivariable analyses (p<0.001). Compared with vaginal delivery, C-section delivery increased the odds of having childhood asthma by 34% (OR 1.34, 95%CI 1.29, 1.39) in unadjusted analysis and 11% adjusting for other exposures of interest and additional confounding covariates (AOR 1.11, 95%CI 1.06, 1.15).

### Infant exposures

**Fig 2:**
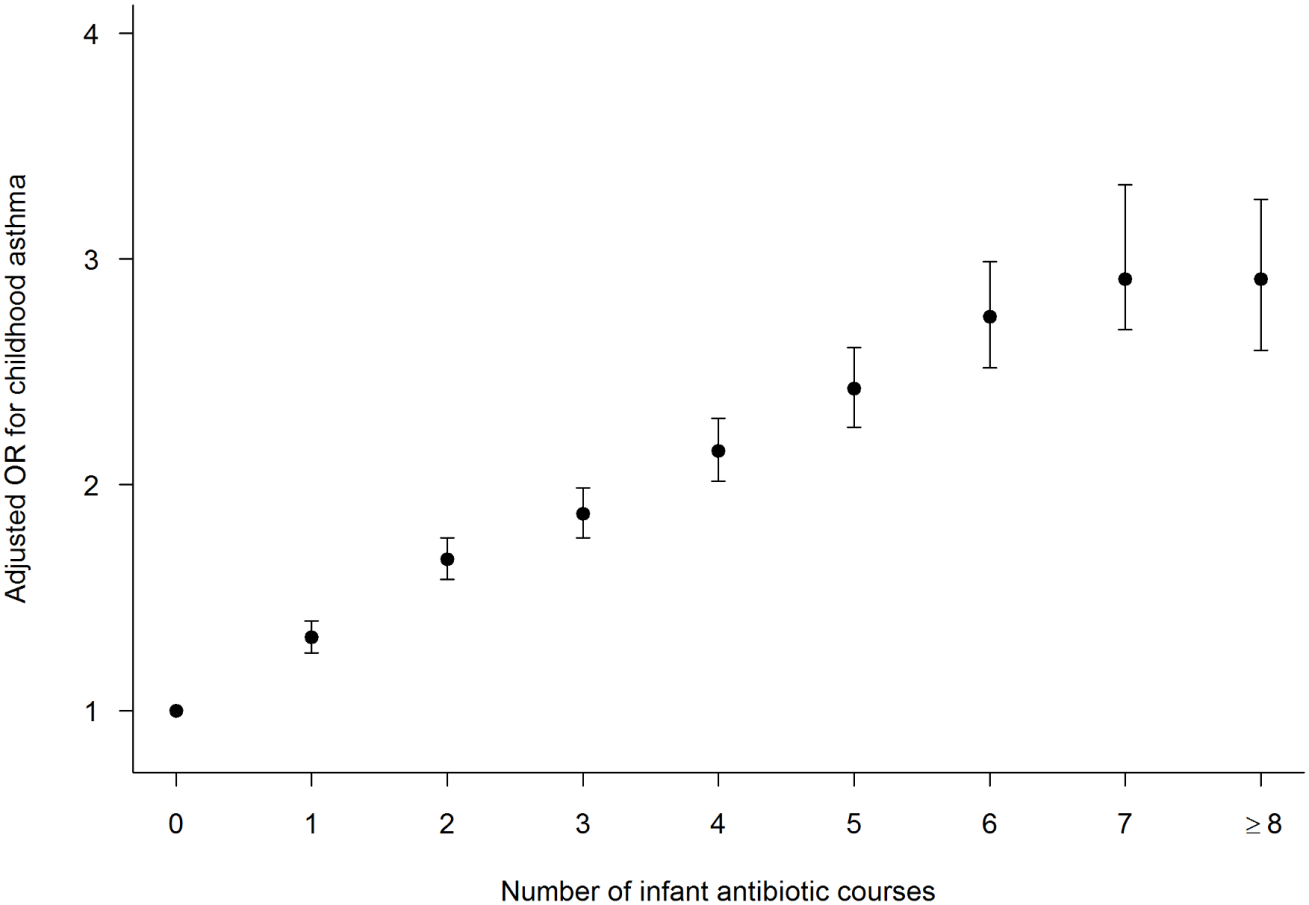
Adjusted odds ratio (AOR) for childhood asthma in relation to the number of antibiotic courses used during infancy. Infants who received no antibiotics during their first 12 months of life served as the reference group.

Compared with children who did not receive any antibiotics in the first year of life, receiving at least one course of antibiotics significantly increased a child’s risk of developing asthma in the unadjusted analysis (OR 2.06, 95%CI 1.98, 2.15). There was a significant dose-dependent relationship between increasing number of infant antibiotic courses and the risk of asthma development. For every additional course of antibiotics, the odds of developing asthma increased by 21% (OR 1.21, 95%CI 1.20, 1.22). This significant dose-dependent relationship between antibiotic use and the risk of childhood asthma persisted after adjusting for other exposures and confounding covariates (AOR 1.16, 95%CI 1.15, 1.17) (Fig 2). In a sensitivity analysis when number of infant infections was used in lieu of the number of antibiotic courses, the dose-response relationship between asthma risk and the number of infections during infancy was similar to that for the number of antibiotic courses (S2 Fig). For each additional infection infants experienced, the odds of having childhood asthma increased by 10% after adjusting for maternal UTI during pregnancy, mode of delivery, number of older siblings and other confounding covariates (AOR 1.10, 95%CI 1.07, 1.13).

**Fig 3:**
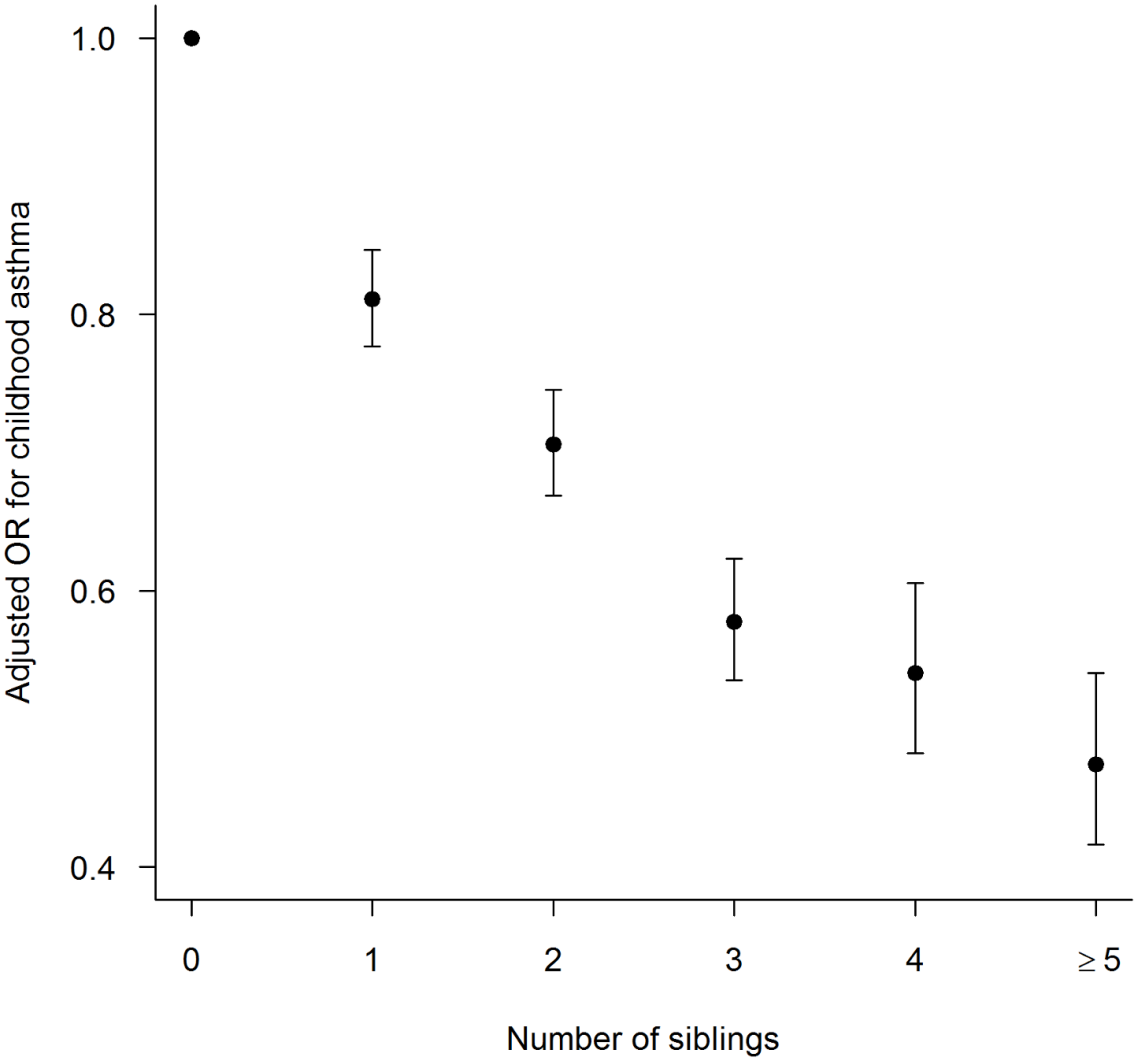
Adjusted odds ratio (AOR) for childhood asthma in relation to the number of older siblings at home. Infants with no older siblings at home served as the reference group.

On the other hand, having older siblings at home significantly decreased the risk of childhood asthma in the unadjusted and after adjusting for other exposures and confounding covariates (Fig 3). There was also a significant dose response relationship between asthma and number of older siblings at home. With every additional sibling at home, the odds of childhood asthma at age 6 years decreased by 8% (OR 0.92, 95%CI 0.91, 0.93) in the unadjusted analysis and 15% after adjusting for other exposures and confounding covariates (AOR 0.85, 95%CI 0.84, 0.87).

### Relative and combined contribution of exposures

The relative contribution and importance of exposures on asthma risk was calculated and plotted as the fraction of variability explained by each exposure (S3 Fig). Compared with known and studied risk factors of childhood asthma, infant antibiotic use conferred the greatest risk, similar to the risk of having a bronchiolitis healthcare visit during infancy. The variability of asthma explained by having older siblings at home ranked next with similar contribution and importance as infant gender and having a mother with asthma.

**Fig 4:**
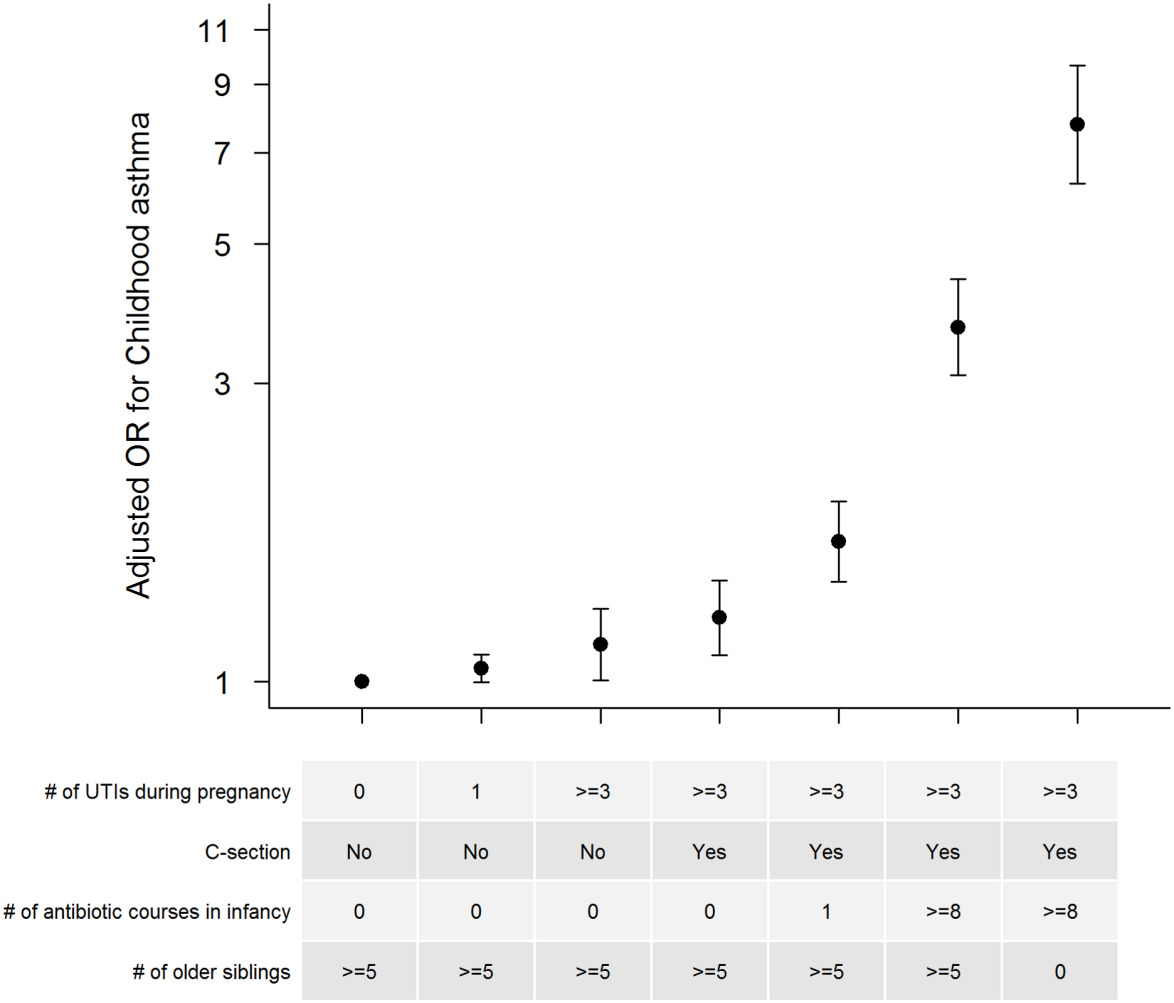
Adjusted odds ratios (AOR) for childhood asthma in various scenarios of cumulative exposures. AOR for childhood asthma in various scenarios of maternal urinary tract infection (UTI) during pregnancy, mode of delivery, infant antibiotic courses, and having older siblings at home. Infants with mothers who did not have a UTI during pregnancy, were born via vaginal delivery, had zero antibiotics during infancy, and had at least five older siblings at home served as the reference group.

We further estimated the combined effects of these events (maternal UTI during pregnancy, mode of delivery, infant antibiotic use, and having older siblings at home) in several different scenarios (Fig 4). Children whose mothers did not have a UTI during pregnancy, were born by vaginal delivery, had at least five older siblings at home, and had not used antibiotics during infancy had the lowest risk of developing asthma and served as reference group. The risk of developing asthma increased with every additional risk factor (Fig 4). In extreme scenarios, children with mothers who had at least three UTIs during pregnancy, were born by C-section, had no older siblings at home, and had used at least eight courses of antibiotics during infancy had a 7.77 fold increase in the odds of developing asthma compared with children with none of these risk factors (AOR 7.77, 95%CI 6.25, 9.65) (Fig 4).

**Fig 5:**
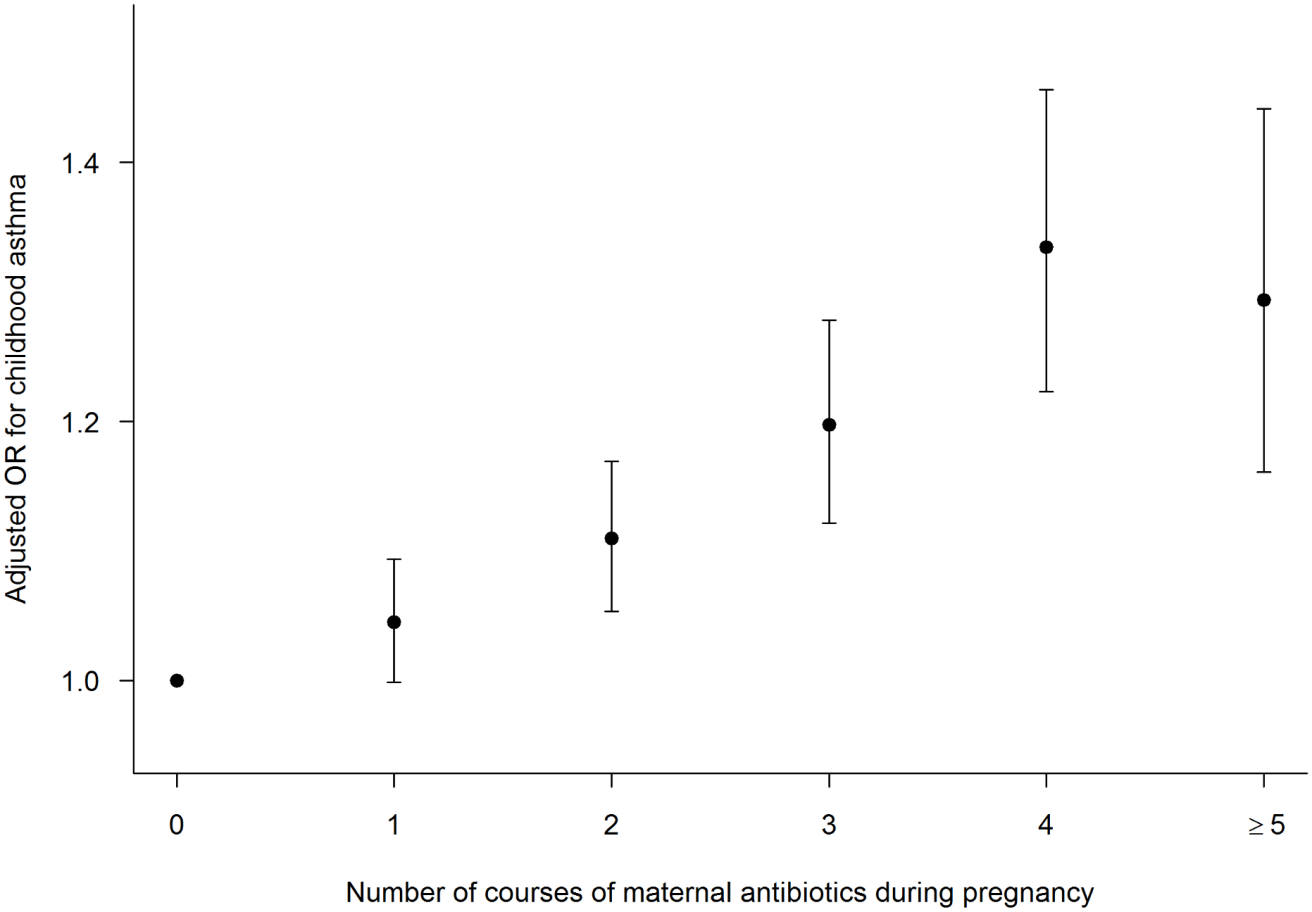
Adjusted odds ratios (AOR) for childhood asthma in relation to the number of antibiotic courses used during pregnancy. Infants with mothers who did not use antibiotics during pregnancy served as the reference group. This is among the group of children who were enrolled in TennCare.

In the subgroup of infants enrolled in TennCare in whom maternal antibiotic dispensing information was available, the number of maternal antibiotic courses (0, 1, 2, 3, 4, 5+) was used in our models. Maternal antibiotic use increased the risk of childhood asthma in a similar dose-dependent manner as did maternal UTI (Fig 5). With each additional antibiotic course during pregnancy, the odds of childhood asthma increased by 13% (OR 1.13, 95%CI 1.12, 1.15) in the unadjusted analysis and 6% (AOR 1.06, 95%CI 1.05, 1.08) after adjusting for mode of delivery, infant antibiotic use, number of older siblings, and other confounding covariates. Lastly, the relationship between exposures of interest with childhood asthma was similar in subgroup analyses of term, non-low birth weight healthy infants and infants who were enrolled in either KPNC or TennCare (data not shown).

## Discussion

In this large population-based cohort study, we reported that *in utero* and early life events, including maternal UTI during pregnancy, maternal antibiotic use, C-section delivery, infant antibiotic use, and having no older siblings at home were all associated with an increased risk of childhood asthma. In addition, there were strong dose-dependent relationships between number of maternal UTIs during pregnancy, maternal antibiotic use, infant antibiotic use, and older siblings at home with asthma risk. Infant exposures including antibiotic use and absence of older siblings at home conferred the greatest risk of childhood asthma development, while maternal exposures such as maternal antibiotic use, UTI, and GBS had less impact on childhood asthma development. Individuals with extremes of multiple exposures had a nearly eight-fold increased odds of developing asthma by age 6 years.

The increased risk of maternal UTI during pregnancy, maternal antibiotic use, mode of delivery, infant antibiotic use, and number of older siblings at home on the development of childhood asthma may act through various mechanisms, one of which may be through altering the maternal and infant microbiome. The human microbiome plays an important symbiotic role in our health. It influences the development of the immune system, and if altered, may lead to an increased risk of diseases such as asthma [3,42]. Recent studies support initial colonization starting in the womb, with supplemental early colonization influenced via delivery route and breast feeding [43]. The maternal genital tract (including the placenta) is likely the main source of the initial microbial colonization, which we and others have demonstrated to significantly differ among infants born via vaginal delivery compared with C-section [19,44,45]. Additionally, the microbiome can be altered by infections such as UTI, and the antibiotics used to treat those infections [46]. Maternal antibiotics are likely to alter the *in utero* microbiome as well as the maternal skin and vaginal flora that provides the sequential colonization at birth [19,47]. Antibiotics can also cross the placenta and stay in the fetal blood stream at high levels for several hours after administration to the mother, which could affect both the *in utero* colonization and that determined by mode of delivery for antibiotics administered perinatally [48]. In our study, maternal UTI during pregnancy and maternal antibiotic use were both associated with an increased risk of childhood asthma in a dose-dependent manner. It is likely that both the infection itself as well as antibiotic use play a separate and additive role in altering the microbiome. In addition, we adjusted for maternal asthma status in the multivariable regression models, demonstrating that antibiotics and infections have an effect that is independent of inherited risk.

As mentioned previously, the mode of delivery leads to divergent bacterial colonization in the infant, which can progress to an altered adult microbiome. If an infant is born via C-section, he or she is largely colonized by skin flora such as *Staphylococcus* and *Corynebacterium*. In contrast, infants’ born via vaginal delivery are colonized with vaginal and fecal flora including *Lactobacillus* and *Prevotella* [19,47]. In agreement with other studies, we found that delivery via C-section, compared to vaginal delivery, increases the risk of developing childhood asthma [49–53].

We also found a significant dose response protective effect of having older siblings on childhood asthma. This significant protective effect may be a reflection of the hygiene hypothesis (as older siblings may share protective bacteria with their infant sibling). Azad and colleagues recently published data from the Canadian Healthy Infant Longitudinal Development (CHILD) birth cohort in which 24 healthy term infants’ intestinal microbiome was measured, demonstrating that having older siblings does alter the microbiome [54].

Our large study population makes it possible for us to tease out the relative impact of early-life exposures in two diverse populations, demonstrating their comparative role, with infant antibiotic use and having fewer older siblings at home having the greatest impact on childhood asthma risk. Infancy is a period of time when infants establish their own microbiome, which influences the longitudinal development of the microbiome, the immune system, and susceptibility to infection, among other roles. While the impact of these risk factors is not likely mediated exclusively through altering the early life microbiome, exposures that potentially alter the microbiome at this period of time may have the greatest impact on both subsequent microbial diversity as well as risk of childhood asthma.

Our study has considerable strengths. First, unlike other studies that have examined all these risk factors individually, we examined the combined effect of relevant exposures in a large a population-based study. Second, we established the effect size of each risk factor, which may help us to understand the most vulnerable period (prenatal, perinatal, or postnatal), and the most important modifiable risk factor in the development of asthma. Third, we were able to demonstrate the dose-dependent association of these exposures and the additive effect of these early life events on asthma risk. Fourth, we measured these exposures prior to the asthma diagnosis. Lastly, we were able to ascertain and adjust for maternal asthma status, an important marker of genetic predisposition to childhood asthma. This study also has important limitations that should be considered. Although we propose that one of the mechanisms through which the exposures measured in this study have an effect on asthma risk is through altering the microbiome, we did not directly measure the microbiome. However, all exposures measured in our study have been previously demonstrated to alter the microbiome [42,44,55–59]. All exposures ascertained and considered in our study have also been demonstrated to be associated with the development of childhood asthma [35,36,49,60–63]. Future studies using next-generation sequencing of the bacterial genome will be important in identifying patterns and components of the microbiome that may be protective or detrimental. To date, studies using these new technologies have had small sample sizes that, unlike ours, have not allowed assessment of the relative effect size, establishment of a dose-dependent relationship, nor ability to assess the combined effect of relevant exposures. Second, we were unable to include several other factors known to alter the early-life microbiome or known to be associated with asthma risk, including the impact of infant’s diet (breast feeding, formula feeding, time of weaning to solids), growing up with pets, early daycare attendance, and other environment exposures [25–28,32,33,35,64–67].

In conclusion, our data show that early-life exposures, maternal antibiotic use /maternal UTI infection, mode of delivery, infant antibiotic use, and number of older siblings at home are associated with childhood asthma development in a cumulative manner, and for those continuous variables, a dose-dependent relationship. Measured infant exposures also had a greater effect than *in utero* and at delivery exposures. The strong association of these exposures with asthma risk, the dose-dependent relationship, and the additive effect suggest that testing interventions to decrease these exposures, perhaps mediated through establishing a protective microbiome, could prevent the development of asthma in children.

## Supporting Information

S1 Fig AOR for childhood asthma in relation to the number of maternal infections during pregnancy. Infants with mothers who did not have an infection during pregnancy served as the reference group.
(PDF)

S2 Fig AOR for childhood asthma in relation to the number of infant infections during the first 12 months of life. Infants with no coded infections during infancy served as the reference group.
(PDF)

S3 Fig Relative contribution and importance of maternal and infant risk factors for childhood asthma. Relative contribution and importance of maternal and infant risk factors as well as other known risk factors for childhood asthma in full penalized model, as judged by the fraction of variability of asthma explained by each exposure.
(PDF)
