# Correction: Relative Importance and Additive Effects of Maternal and Infant Risk Factors on Childhood Asthma

**DOI:** 10.1371/journal.pone.0156473

**Published:** 2016-05-24

**Authors:** Pingsheng Wu, Amy S. Feldman, Christian Rosas-Salazar, Kristina James, Gabriel Escobar, Tebeb Gebretsadik, Sherian Xu Li, Kecia N. Carroll, Eileen Walsh, Edward Mitchel, Suman Das, Rajesh Kumar, Chang Yu, William D. Dupont, Tina V. Hartert

There is an error in the first sentence of the conclusion section of the abstract. The correct sentence is: Early-life exposures, maternal UTI during pregnancy (maternal antibiotic use), mode of delivery, infant antibiotic use, and having no older siblings at home, are associated with an increased risk of childhood asthma in a cumulative manner, and for those continuous variables, a dose-dependent relationship.
