# Supplementary figures and images for: Relative Importance and Additive Effects of Maternal and Infant Risk Factors on Childhood Asthma
Source: PLoS One. 2016 Mar 22;11(3):e0151705. doi: 10.1371/journal.pone.0151705 (PMC4803347; doi:10.1371/journal.pone.0151705)

S1 Fig.

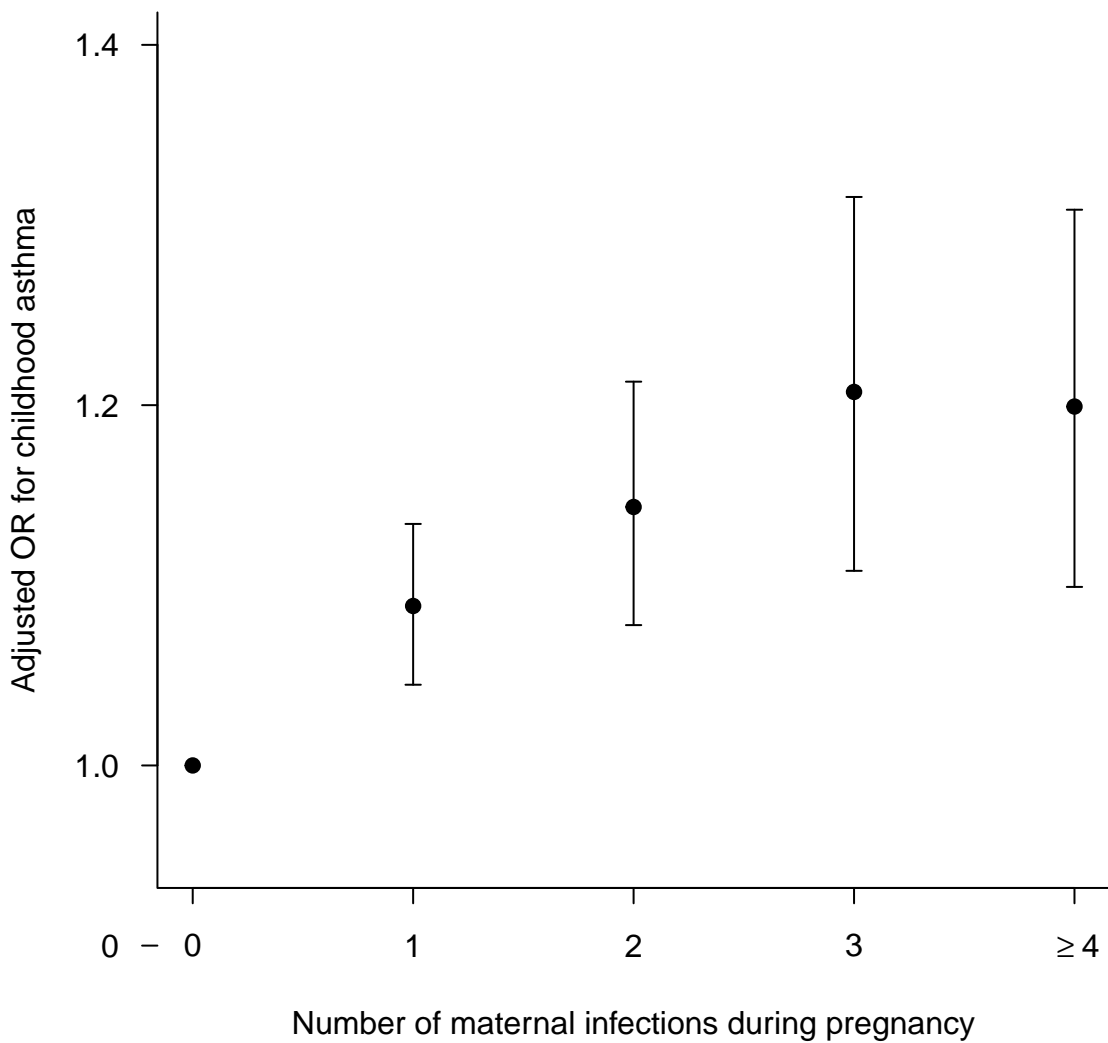

Supplement: S1 Fig — Infants with mothers who did not have an infection during pregnancy served as the reference group. (PDF) [file pone.0151705.s001.pdf]

S2 Fig.

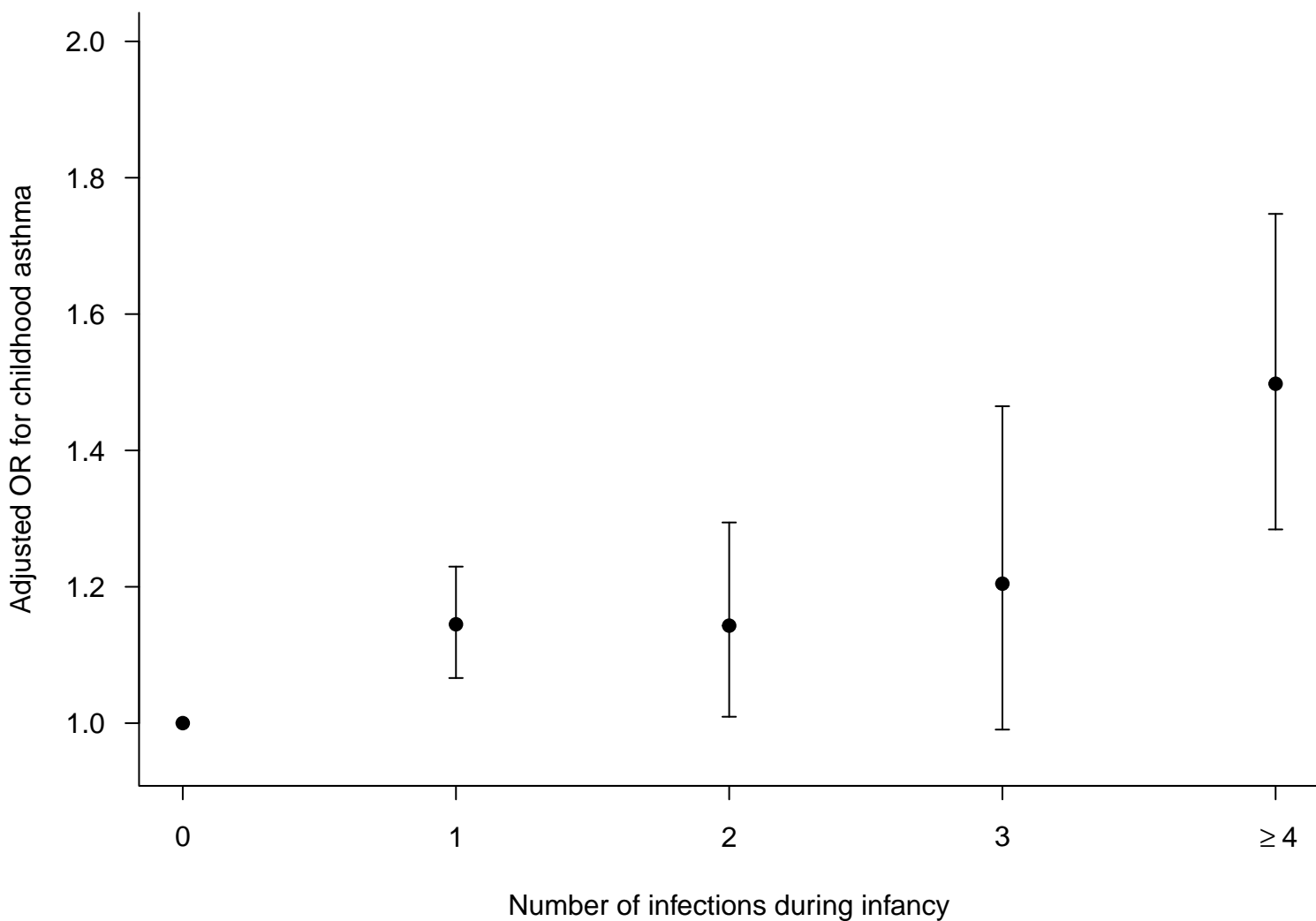

Supplement: S2 Fig — Infants with no coded infections during infancy served as the reference group. (PDF) [file pone.0151705.s002.pdf]
