# Supplementary material for: Relative Importance and Additive Effects of Maternal and Infant Risk Factors on Childhood Asthma
Source: PLoS One. 2016 Mar 22;11(3):e0151705. doi: 10.1371/journal.pone.0151705 (PMC4803347; doi:10.1371/journal.pone.0151705)
Supplement: S3 Fig — Relative contribution and importance of maternal and infant risk factors as well as other known risk factors for childhood asthma in full penalized model, as judged by the fraction of variability of asthma explained by each exposure. (PDF) [file pone.0151705.s003.pdf]

S3 Fig.

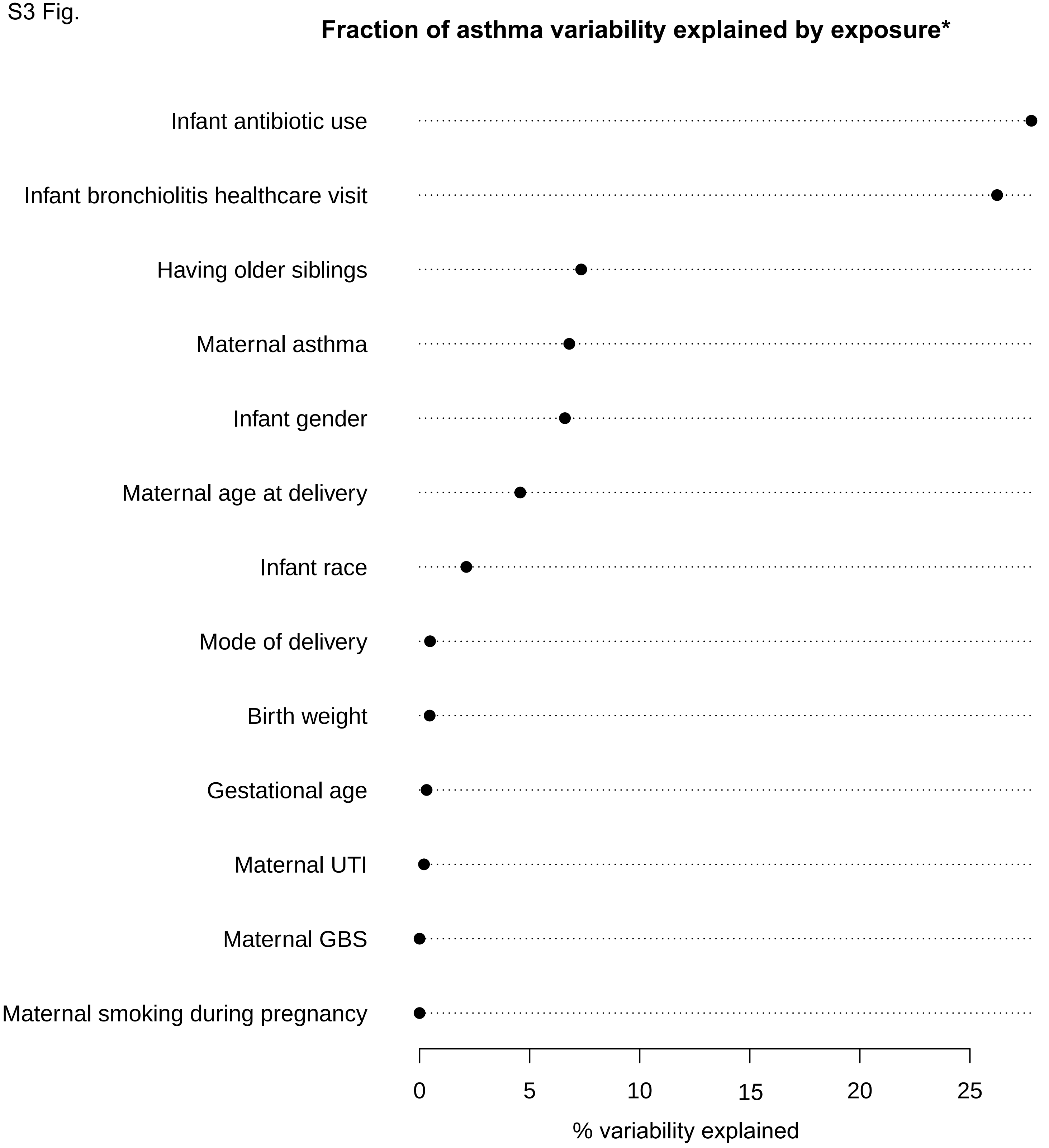

*Additional risk factors that are not plotted include: birth hospitalization length of stay, birth year, birth month, having chronic lung disease, having congenital heart disease, maternal education, and study site
